# Quality Care Assessment of Maternal and Child Health Services Among Women in Sagar City: A Cross-Sectional Study

**DOI:** 10.7759/cureus.83033

**Published:** 2025-04-26

**Authors:** Shefali Jain, Amarnath Gupta, Shraddha Mishra, Bhupendra K Rohit

**Affiliations:** 1 Preventive Medicine, Government Medical College, Satna, Satna, IND; 2 Preventive Medicine, Government Bundelkhand Medical College, Sagar, IND; 3 Community Medicine, Government Bundelkhand Medical College, Sagar, IND

**Keywords:** antenatal care, intranatal care, knowledge, maternal and child health, postnatal care, quality of care

## Abstract

Background: Ensuring high-quality maternal and newborn healthcare is crucial for improving health outcomes in low- and middle-income countries such as India. This study aimed to assess the quality of maternal and child health (MCH) services in Sagar city, Madhya Pradesh, India, and their associations with beneficiaries' knowledge.

Methods: A cross-sectional study was conducted among 400 postnatal women. Data on sociodemographic characteristics, obstetric history, and MCH service utilization were collected. A scoring system was developed to evaluate the quality of MCH services, including antenatal care (ANC), intranatal care (INC), and postnatal care (PNC).

Results: While most women were aware of early registration, institutional delivery, and breastfeeding benefits, knowledge gaps existed in areas such as ANC visits, TT immunization, and exclusive breastfeeding. Only 25 (6.25%) of the participants received good-quality care, whereas 201 (50.25%) received poor-quality care. A significant association was found between the knowledge score and quality of care (ꭕ²=43.131, p=0.000).

Conclusion: This study highlights the need for interventions to improve knowledge and awareness about MCH services among beneficiaries and to increase the quality of care provided. Improving the quality of MCH services is essential for reducing maternal and child mortality and achieving better health outcomes in India.

## Introduction

The quality of care in Maternal and Child Health (MCH) refers to providing safe, effective, respectful, and timely healthcare to mothers and children to ensure positive health outcomes. Even in 2024, as we reviewed progress towards the Millennium Development Goals (MDGs), despite significant progress in reducing mortality, we still have unacceptably high numbers of maternal and newborn deaths globally [1]. Against this background, governments and donors are exploring ways to reduce cost barriers for pregnant women, and many demand-side financing schemes designed to stimulate demand for maternal health care have been implemented globally [2,3]. High-quality MCH care includes access to skilled health personnel, essential medicines, antenatal and postnatal services, and well-functioning referral systems. High-quality maternal and newborn healthcare is essential to improve mothers' and babies' experiences and health outcomes [4,5]. In many low- to middle-income countries, such as India, there are initiatives to increase antenatal care attendance and facilitate births [6,7]. Emphasizes the Quality of Care (QoC) as “the extent to which health care services provided to individuals and patient populations improve desired health outcomes” [8,9]. While several QoC frameworks exist in the literature, not all provide a blueprint for approaching the QoC as a multidimensional construct [8,10]. To develop and implement appropriate, relevant, and contextualized initiatives for a community, it is important to understand how the quality of care is perceived and defined by different maternal and newborn healthcare stakeholders [11]. WHO envisages that ‘every pregnant woman and newborn will receive quality care throughout pregnancy, childbirth, and the postnatal period’ under the umbrella of Universal Health Coverage (UHC), which is aligned with ‘Ending Preventable Maternal Mortality’ and the ‘Every Newborn Action Plan’ [12]. Improving quality of care has become increasingly important as part of low- and middle-income countries’ (LMICs) efforts to achieve the Sustainable Development Goals (SDGs) [13,14]. Reducing maternal, neonatal, and infant mortality is a critical issue in many LMICs; reducing maternal and child mortality is the highest priority of the United Nations Sustainable Development Goals among its health-related targets [15]. Around the world, health systems fail to provide good QoC. By developing learning systems, health systems can better identify good practices and explain how to sustain and scale these good practices [16]. Evidence has emerged from diverse settings that increasing facility delivery may not reduce mortality if the QoC is poor [17]. Poor QoC impacts individual health outcomes and increases risks and costs for the entire community [18]. As facility deliveries increase in developing countries, there are concerns about service quality [17]. Despite the decreasing maternal mortality ratio (MMR) and neonatal mortality rate (NMR) and increasing rates of facility deliveries, an estimated 303,000 maternal deaths, 2.7 million neonatal deaths, and 2.6 million stillbirths occurred in 2015 worldwide [19]. As more people expect healthcare to focus on their needs, and as health outcomes and disease priorities change in LMICs, there is a greater need for high-quality health systems. Since our country aims to achieve UHC by 2030, simply providing access to healthcare is not enough; it must also be effective, safe, trustworthy, and centered around patients. A good health system should be fair, strong, and efficient. When it comes to antenatal care (ANC), quality of healthcare is essential. However, there are no widely accepted ways to measure the quality of ANC in low-resource settings. So far, only one tool has been tested for reliability, and that was in a high-resource setting. Without a standardized way to measure quality, researchers have used different combinations of factors to create their measures. Since process-based measures are easier to track, they are often used as substitutes for assessing quality [20]. Among the few studies concerning the quality of health care in sub-Saharan Africa, the majority have created a simple quality index using unweighted averages of a combination of ANC variables. Although simple to implement, equal weighting constrains each variable to be as important as each other and does not adjust for correlations between variables [21]. Although good quality of care is a multidimensional notion and there are no universally agreed-upon indicators for assessing the quality of health care [22], in this study, we assess the quality of care by assessing some components of ANC (early registration, number of visits, iron folic acid [IFA] tablets, tetanus toxoid [TT] immunization), Intranatal care (INC) (place of delivery), and postnatal care (PNC) (number of postnatal visits) and determine its association with the mother’s knowledge of maternal and child health services. This study emphasizes continuity of care from pregnancy through childhood.

## Materials and methods

Study design and setting

This cross-sectional study was conducted between March 2020 and October 2021 in Sagar City, Madhya Pradesh, India, to assess the quality of maternal and child health (MCH) services among postnatal women.

Sample size and sampling technique

The sample size was determined based on the prevalence of antenatal check-ups in the first trimester, as reported in the National Family Health Survey (NFHS-4), using a 10% allowable error and a 95% confidence level. This yielded a sample size of 400 postnatal women. Of these, 200 women (50%) were selected from health institutions using convenient sampling. Participants were recruited from four health facilities: the District Hospital, the Medical College, and two Urban Primary Health Centers (UPHCs). Immunization clinics were visited mostly on immunization days, and 10 women were selected randomly per session who had brought their newborns for the 0-dose vaccination. These women were contacted telephonically after three weeks and interviewed to assess their postnatal care practices. The remaining 200 women (50%) were selected through simple random sampling from 20 Anganwadi Centers, randomly chosen out of 100 in Sagar City. Ten beneficiaries from each selected center were identified from center records, and ASHA workers assisted in locating them for home interviews. Repeat visits were made to reach participants who were not available during the initial visit.

Ethical considerations

Ethical approval for the study was granted by the Institutional Ethical Committee of Bundelkhand Medical College, Sagar, Madhya Pradesh. The approval was obtained under the New Drug and Clinical Trial Rules, 2019, with Approval Certificate No. IECBMC/2020/07.

Data collection procedure

Data were collected through face-to-face interviews using a structured and pretested questionnaire. The tool included sections on sociodemographic characteristics such as age, education, occupation, religion, family size, and monthly income. It also captured obstetric history, including age at first pregnancy, number of pregnancies, live births, abortions, stillbirths, neonatal deaths, and birth spacing. Utilization of MCH services was assessed across antenatal, intranatal, and postnatal care components. Antenatal care indicators included early registration, number of ANC visits, intake of iron-folic acid (IFA) tablets, and tetanus toxoid (TT) immunizations. Intranatal care covered the place and type of delivery and birth weight. Postnatal care aspects included the timing of breastfeeding initiation, immunization status, and number of postnatal visits.

Assessment of MCH service utilization and quality of care

A scoring system was used to evaluate service utilization. Antenatal care was assessed through four parameters: early registration within 12 weeks, a minimum of four ANC visits, consumption of at least 100 IFA tablets, and two TT injections or a booster dose. Each fulfilled parameter was assigned a score of 1, and non-fulfillment scored 0. A total score of 4 indicated complete utilization, scores of 1-3 were categorized as underutilization, and a score of 0 denoted no utilization. Intranatal care was evaluated based on whether the delivery occurred at a healthcare institution by a skilled provider, scoring 1 for institutional and 0 for home delivery. Postnatal care was considered adequate if the mother had at least three postnatal visits, scoring 1; otherwise, 0.

The quality of care (QoC) for ANC, INC, and PNC was also measured using a separate scoring scale. Each quality-related activity received a score of 1 if performed and 0 if not. The total QoC score was calculated for each service area. An average score was derived for overall quality of care, where a score of 5 or more was categorized as good, 4 as average, and 3 or less as poor. The maximum possible score for overall QoC was 6.

Data quality and statistical analysis

To ensure high data quality, data collectors underwent rigorous training, and a pilot study was conducted to pretest the questionnaire. Double data entry was used for all critical variables to minimize errors. Descriptive statistics were applied to summarize the data. Additionally, the chi-square test was used to assess associations between the beneficiaries’ knowledge scores and their corresponding quality of care scores.

## Results

Most participants in the study were women in the age group 20-25 years, 198 (49.5%), followed by women age group 26-30 years, 120 (30.0%). Most participants had a senior secondary education, 156 (39.0%), followed by primary school, 80 (20.0%). The majority were Hindu, 298 (74.5%), followed by Muslim, 55 (13.8%). Joint families were the most common (194 [48.5%]), followed by nuclear families 194 (34.0%). The most common per capita income range was 2253-3808, i.e., 96 (24.0%), followed by 3808-7769, i.e., 76 (19.0%) (Table 1).

**Table 1:** Sociodemographic profile of the study beneficiaries (mothers)

Variables	Characteristics	Number of Beneficiaries	Percentage
Age of mother	<20 years	25	6.3
	20-25 years	198	49.5
	26-30 years	120	30.0
	>30 years	57	14.3
Education of mother	Illiterate	150	37.5
	Primary School	80	20.0
	Senior secondary	156	39.0
	Higher education	14	3.5
Occupation of mother	Unemployed/Homemaker	286	71.5
	Unskilled, skilled Worker, Clerical, Shop Owner, Farmer	104	26.0
	Profession	10	2.5
Type of Family	Nuclear	136	34.0
	Joint	194	48.5
	Three generation	70	17.5
Religion of mother	Hindu	298	74.5
	Muslim	55	13.8
	Others	47	11.8
Socio-economic class	7770 & above	80	20.0
	3808-7769	76	19.0
	2253-3808	96	24.0
	1166-2253	88	22.0
	<1166	60	15.0

A study on knowledge about maternal and child health services among beneficiaries in Sagar, Madhya Pradesh, India, revealed mixed findings. While a significant majority of respondents were aware of early registration, i.e., 307 (76.75%); institutional delivery, i.e., 308 (77%); and the benefits of early initiation of breastfeeding, i.e., 339 (84.75%), knowledge gaps were evident in areas such as minimum ANC visits, i.e., 188 (47%); TT immunization completion, i.e., 184 (46%); and exclusive breastfeeding, i.e., 89 (22.25%) (Table 2). The distribution of beneficiaries based on knowledge scores revealed that 77 (19.25%) had good knowledge, 103 (25.75%) had average knowledge, and 220 (55%) had poor knowledge (Table 3). These results highlight the need for targeted interventions to improve knowledge and awareness about maternal and child health services among the study population.

**Table 2:** Awareness and knowledge levels of beneficiaries on maternal and child health practices

Knowledge regarding	Number of Beneficiaries	Percentage
Early registration	307	76.75
Minimum 4 ANC visit	188	47
Importance of TT immunization	146	36.5
TT immunization complete dose	184	46
Importance of iron and folic acid tablets	176	44
Quantity of iron and folic acid tablets should be consumed in pregnancy	130	32.5
Supplementary nutrition available in Anganwadi	202	50.5
Importance of Institutional Delivery	308	77
Janani express available for transport to hospital for institutional delivery	189	47.25
JSY cash incentive in institutional delivery	308	77
Awareness about early initiation of breast feeding	288	72
Benefits of early initiation of breast feeding	339	84.75
Exclusive breast feeding and avoid prelacteal feeding	89	22.25
Benefits of immunization of infant	280	70
No. of Postnatal visit	52	13

**Table 3:** Knowledge assessment scores of beneficiaries on maternal and child health practices

Knowledge of Beneficiaries (Score Maximum=15)	No. of beneficiaries	Percentage
Good [≥12]	77	19.25
Average [9-11]	103	25.75
Poor [<9]	220	55
Total	400	100

**Figure 1:**
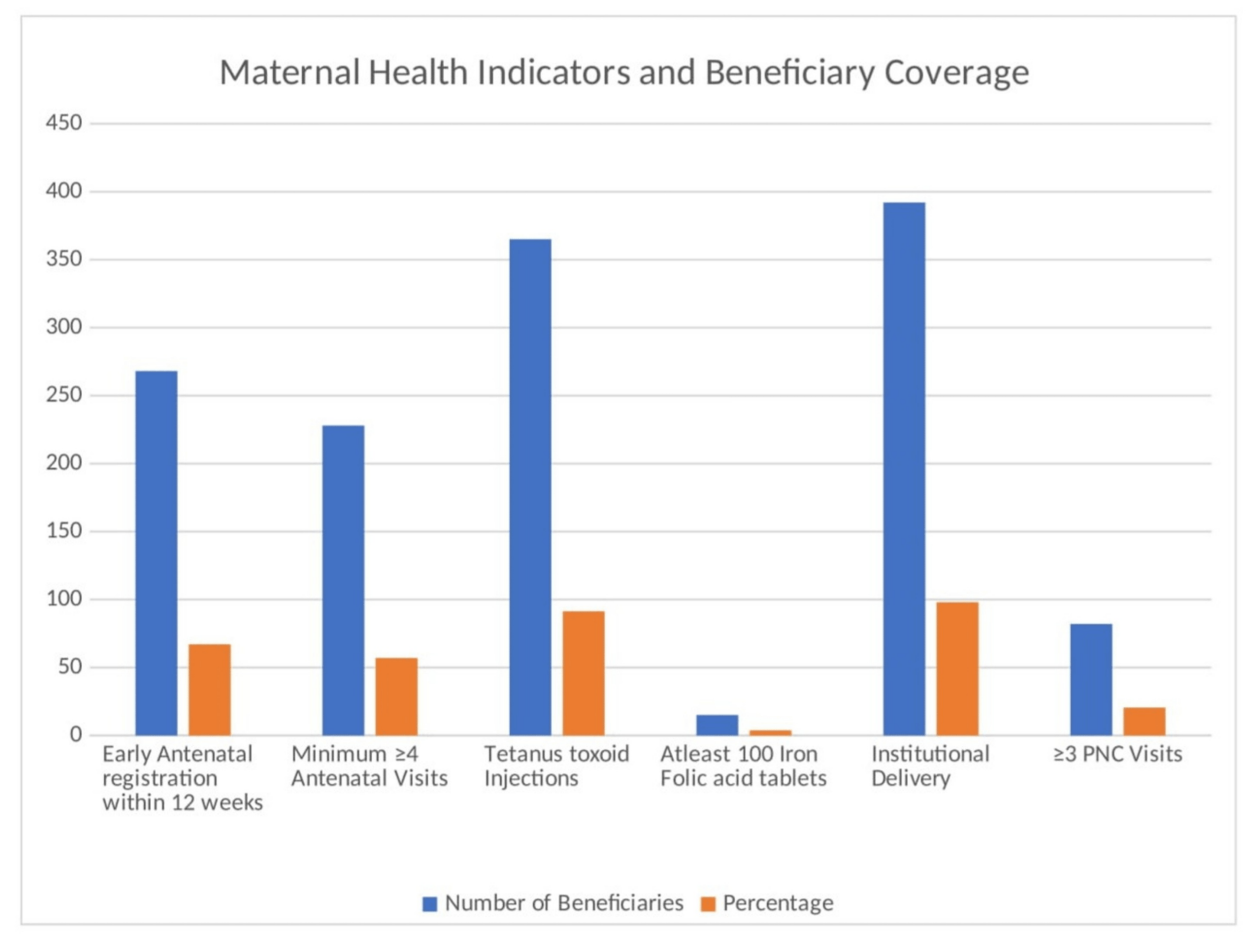
Distribution of beneficiaries maternal health indicators

**Figure 2:**
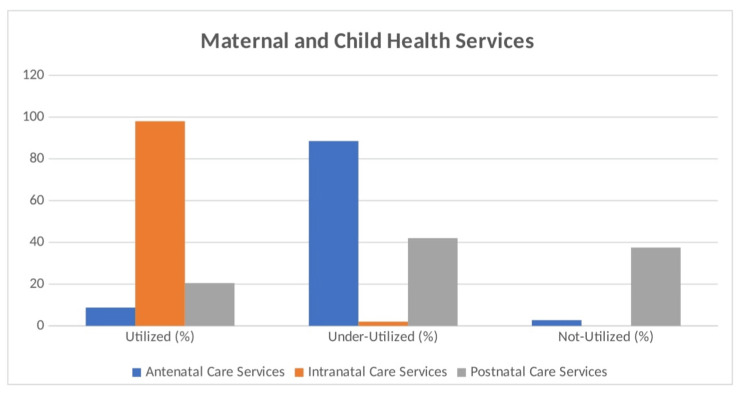
Distribution of beneficiaries according to MCH care services utilization MCH: Maternal and child health

The maternal and child health services report shows the following coverage among beneficiaries: 268 (67%) registered early for antenatal care within 12 weeks, 228 (57%) had at least four antenatal visits, and 365 (91.25%) received tetanus toxoid injections. Only 15 (3.75%) of the participants received at least 100 iron folic acid tablets. All deliveries were either institutional or assisted by trained staff, and 82 (20.5%) had three or more postnatal care visits (Figure 1). The utilization of maternal and child health services reveals varying degrees of engagement. Antenatal care services were significantly underutilized, with only 8.75% of beneficiaries utilizing them, whereas 354 (88.5%) underutilized them and 11 (2.75%) did not utilize these services at all. In contrast, intranatal care services were fully utilized, with 400 (100%) engagements. Postnatal care services showed a more mixed picture: 82 (20.5%) utilized the services, 168 (42%) underutilized them, and 150 (37.5%) did not utilize them at all (Table 4; Figure 2). This study evaluated the perceived quality of maternal and child health services among 400 beneficiaries, categorized as good (6.25%), average (43.5%), and poor (50.25%). A total of 201 (50.25%) of the respondents rated the care as poor, indicating significant dissatisfaction, whereas 25 (6.25%) reported that the services were excellent (Table 5).

**Table 4:** Utilization, underutilization, and non-utilization of maternal and child health services

Maternal and Child Health Services	Utilized	Utilized (%)	Underutilized	Underutilized (%)	Not-utilized	Not-utilized (%)
Antenatal Care Services	35	8.75	354	88.5	11	2.75
Intranatal Care Services	400	100	0	0	0	0
Postnatal Care Services	82	20.5	168	42	150	37.5

**Table 5:** Quality of care assessment scores among beneficiaries

Quality of Care	Score	Percentage
Good [≥5]	25	6.25
Average [=4]	174	43.5
Poor [≤3]	201	50.25
Total	400	100

The chi-square test with 4 degrees of freedom (df=4) revealed a significant association between the knowledge score and quality of care (ꭕ²=43.131, p=0.000) (Table 6). This suggests that there is a strong relationship between the level of knowledge and the perceived quality of care provided. Specifically, individuals with higher knowledge scores tend to rate the quality of care better than those with lower knowledge scores. These findings highlight critical gaps in service delivery and the need for improvements in healthcare quality, staff training, and resource availability. Addressing these issues can enhance beneficiary satisfaction and overall care outcomes.

**Table 6:** Association of knowledge score of beneficiaries with quality of care of MCH services MCH: Maternal and child health

Knowledge score	Quality of Care				
	Good	Average	Poor	Total	df=4 ꭕ^2^=43.131 p=0.000
Good Score	14	72	30	116	
Average Score	2	35	46	83	
Poor Score	9	67	125	201	
Total	25	174	201	400	

## Discussion

This study provides a comprehensive assessment of the quality of maternal and child health (MCH) services in Sagar City, Madhya Pradesh, India, and explores its association with beneficiaries’ knowledge levels. The findings underscore critical gaps in service utilization, knowledge, and quality, which collectively influence maternal and neonatal health outcomes.

The knowledge levels of the study participants were concerning, with 220 (55%) categorized as having poor knowledge. A similar study done in Ethiopia revealed that 81.4% of mothers had poor knowledge of maternal healthcare services [5]. Although in our study, a significant proportion of women were aware of key aspects such as early registration (307 [76.75%]), institutional delivery (308 [77%]), and early breastfeeding initiation (339 [84.75%]), knowledge gaps were notable in areas critical to optimal care, such as completing at least four ANC visits (188 [47%]), TT immunization (184 [46%]), and exclusive breastfeeding (89 [22.25%]). The low awareness of ANC components is particularly alarming, as antenatal care is the foundation for ensuring maternal and neonatal health. Similar studies have shown that incomplete or delayed antenatal care can lead to adverse outcomes, including undetected pregnancy complications and higher maternal mortality rates [5,23].

The service utilization data revealed a striking contrast between high institutional delivery rates (400, 100%) and poor ANC and PNC utilization. While the universal institutional delivery rate aligns with government programs promoting facility-based births, the low utilization of postnatal services (82, or 20.5%) and poor adherence to recommended ANC practices (only 35, or 8.75%, achieved complete utilization) highlight gaps in service delivery and accessibility. A similar study done in Ethiopia reported that 44.5% of mothers had at least four visits (ANC-4) during their recent pregnancy [5]. In another study, the results were that 51.6% had 4 or more ANC visits [24]. A similar study done in Jabalpur found that 58.80% of women received four or more antenatal visits [25] and 61% reported three or more ANC visits [26]. A total of 398 (99.7%) of the women were vaccinated with tetanus toxoid [TT]. Similarly, another study found that 79.6% had TT immunization during pregnancy [27].

In this study, 72 (17.8%) of women had received at least one PNC service within six weeks after delivery. This mismatch may reflect systemic barriers such as inadequate health education. A similar study done to assess the knowledge regarding antenatal, intranatal, and postnatal services observed that only 55% of study subjects were aware of antenatal services, whereas only 30% of women knew about postnatal services. A total of 38% of the participants were aware of institutional delivery, and 58% of the women knew about home delivery by a trained person. In that study also, there was a lack of knowledge regarding MCH services among the study participants, which led to the underutilization of services, variability, and cultural practices that deprioritize routine check-ups post-delivery [28]. The overall quality of care (QoC) ratings indicated significant dissatisfaction, with 201 (50.25%) of the respondents rating care as poor and only 25 (6.25%) categorizing it as good. The low QoC scores across ANC, INC, and PNC services emphasize the challenges faced by the healthcare system in maintaining consistent service standards. Factors influencing poor ratings likely include inadequate infrastructure, staff shortages, supply chain inefficiencies (e.g., only 15 [3.75%] received 100 IFA tablets), and a lack of person-centered care, which aligns with findings in other LMIC settings.

The strong association between knowledge scores and perceived QoC (ꭕ²=43.131, p=0.000) underscores the role of health education in shaping healthcare experiences. Women with greater knowledge were more likely to utilize services and report better care experiences, likely because of their ability to advocate for themselves and navigate the healthcare system effectively. This highlights the critical need to strengthen community-based educational programs, leveraging platforms such as Anganwadi centers and ASHA workers to disseminate accurate information on MCH services. Another study reported that women who had good knowledge were 9.88 times more likely to utilize a continuum of maternal healthcare services than women who had poor knowledge of MCH services [5].

The findings of this study underscore the need for targeted policy and programmatic interventions to enhance maternal and child health outcomes in India. Strengthening ANC and PNC services should be a priority, ensuring the availability of essential interventions such as IFA supplementation, TT immunization, and regular postnatal follow-ups. Mobile health technologies and home-based PNC programs could help bridge service access gaps. Community-based awareness campaigns should be expanded to educate women on the importance of ANC visits, exclusive breastfeeding, and maternal immunization. Leveraging community platforms such as Anganwadi centers and ASHA workers can improve outreach and cultural acceptance of health education initiatives. Enhancing the quality of MCH services will require substantial investments in healthcare infrastructure, workforce capacity building, and patient-centered care approaches. Additionally, integrating various government health initiatives, such as Janani Suraksha Yojana (JSY) and Mission Indradhanush, could optimize service delivery and improve maternal and child health outcomes.

Despite its valuable insights, the study has limitations that should be acknowledged. The reliance on self-reported data introduces potential recall and response biases, which may impact the accuracy of service utilization and satisfaction findings. Moreover, as the study was conducted in Sagar City, its findings may not be fully generalizable to other regions with different socio-cultural and healthcare dynamics. Also, the study was conducted during the COVID-19 era, so there are various shortcomings, like transportation, unavailability of appropriate services, etc., that cannot be neglected as they also play a crucial role in affecting the results. Future research should explore the perspectives of healthcare providers and policymakers to identify systemic barriers to improving service quality. Longitudinal studies would also be beneficial in assessing the impact of targeted interventions over time. Addressing these gaps through evidence-based policymaking and community-driven approaches will be critical in advancing maternal and child health in India.

## Conclusions

The findings of this study highlight significant gaps in the knowledge, utilization, and quality of maternal and child health services in Sagar City. Bridging these gaps requires a multipronged approach that addresses both demand-side barriers, such as low awareness and education, and supply-side issues, including inadequate service delivery and infrastructure. By prioritizing knowledge dissemination and enhancing the quality of care, policymakers and healthcare providers can make significant strides in improving maternal and child health outcomes, ultimately contributing to the achievement of sustainable development goals.
